# An FGFR1-Binding Peptide Modified Liposome for siRNA Delivery in Lung Cancer

**DOI:** 10.3390/ijms23158380

**Published:** 2022-07-29

**Authors:** Zhipeng Dong, Yunxue Yin, Jun Luo, Bingxia Li, Fangning Lou, Qiyan Wang, Qingfa Zhou, Baofen Ye, Yue Wang

**Affiliations:** Key Laboratory of Biomedical Functional Materials, School of Sciences, China Pharmaceutical University, Nanjing 211198, China; dzp_hj@126.com (Z.D.); yin18263771356@126.com (Y.Y.); lj_0801@126.com (J.L.); tiebingxia@163.com (B.L.); ninglfn@163.com (F.L.); 18168066085wqy@gmail.com (Q.W.); zqf@mail.ustc.edu.cn (Q.Z.)

**Keywords:** CP7, liposome, target delivery, FGFR1, gene drug

## Abstract

Liposome modification by targeting ligands has been used to mediate specific interactions and drug delivery to target cells. In this study, a new peptide ligand, CP7, was found to be able to effectively bind to FGFR1 through reverse molecular docking and could cooperate with VEGFR3 to achieve targeting of A549 cells. CP7 was modified on the surface of the liposome to construct a targeted and safe nanovehicle for the delivery of a therapeutic gene, Mcl-1 siRNA. Due to the specific binding between CP7 and A549 cells, siRNA-loaded liposome-PEG-CP7 showed increased cellular uptake in vitro, resulting in significant apoptosis of tumor cells through silencing of the Mcl-1 gene, which is associated with apoptosis and angiogenesis. This gene delivery system also showed significantly better antitumor activity in tumor-bearing mice in vivo. All of these suggested that siRNA-loaded liposome-PEG-CP7 could be a promising gene drug delivery system with good bioavailability and minimal side effects for treatment.

## 1. Introduction

With the development of genetic engineering [1], cell engineering [2], and protein engineering [3], molecular biology has shown a leading position in life science [4]. Owing to breakthroughs in related research, gene therapy occupies an important status in the diagnosis and treatment of diseases and has been increasingly studied in the field of biomedicine [5,6,7].

Unlike small-molecule drugs and biological agents that control cancer by inhibiting mutant proteins, RNA interference (RNAi) blocks the production of disease-causing proteins by translation of certain genes, leading to a new gene-targeting technique that can directly block the occurrence of source diseases [8,9]. More and more siRNA drugs have entered the clinical trial phase continuously since the siRNA drug CALAA-01, the first clinical case of gene therapy for solid tumors of the Calando Pharmaceuticals company was approved by the FDA in 2008 [10].

It is noticeable, however, that many defects of siRNA, such as poor cell membrane permeability, lack of targeted effects, rapid degradation by ubiquitous RNase in serum, and removal by the liver and kidneys, would result in unsatisfactory therapeutic effects [11,12]. In 2015, the GalNAc-siRNA conjugate using the sialoglycoprotein receptor uptake pathway of liver cells to achieve the targeted delivery of RNAi drug patisiran was developed by Alnylam for the treatment of rare disease familial amyloid polyneuropathy (FAP) patients with transthyretin-associated amyloidosis (ATTR), which was finally approved in 2018, later becoming the first RNAi drug to be marketed [13]. Therefore, the development of an effective, safe, and stable delivery system is critical to the clinical application of siRNA. In the last few decades, many attempts have been made to develop delivery systems for siRNA [14,15].

In terms of the characteristics of siRNA drugs, we know that the main approach is to functionalize gene carriers. Owing to their good biocompatibility and biodegradability, as well as low toxicity and immunogenicity, liposomes have received increasing attention in this field [16,17,18]. In order to enhance the therapeutic effect, molecules capable of transmembrane targeting have been designed to achieve efficient targeted delivery of siRNA drugs [19].

CIQPFYP (CP7) is a novel peptide designed and screened by our group, which can efficiently combine with VEGFR-3 [20]. In a successive study, we found that CP7-PEG-b-PLL/DOX showed better inhibitory activity on A549 tumor models both in vitro and in vivo than DOX alone, which is obviously due to the excellent targeting effect of CP7 to A549 cells [21]. These experimental results in turn inspired us to further explore the reasons for CP7’s high targeting ability to A549 cells. Here, we made a hypothesis that there may be other receptors on A549 cells that allow effective binding of CP7. Based on this assumption, four transmembrane proteins that have been reported to be overexpressed on A549 cells, including VEGFR1 (PDB Code: 5T89), VEGFR2 (PDB Code: 5OYJ), EGFR (PDB Code: 4KRL), and FGFR1 (PDB Code: 1EVT), were selected as potential targets for further study [22]. The reverse molecular docking of these proteins and CP7 was performed by Schödinger 2018 software. Through hydrogen bond interaction between ligand and receptor protein and scoring poses of CP7 in selected receptor-protein-specified binding sites, we found that CP7 had a better interaction with FGFR1 than others mentioned above.

It is known that an appropriate type of siRNA will directly determine the treatment effect for certain diseases. Many studies have shown that endogenous ligand binding to FGFR1 activates the PI3K/Akt/mTOR signaling pathway and upregulates myeloid cell leukemia 1 (Mcl-1), which is an antiapoptotic protein involved in the regulation of apoptosis, differentiation, and cell cycle in many cell lines, making it crucial for cell survival and growth [23,24,25]. Overexpression of Mcl-1 is closely related to tumorigenesis such as leukemia, non-small-cell lung cancer, breast cancer, and ovarian cancer [26]. Therefore, by using the targeting effect of CP7 to deliver Mcl-1 siRNA to A549 cells, the overexpressed Mcl-1 will be inhibited, followed by the promotion of tumor cell apoptosis and the inhibition of tumor growth [27].

**Scheme 1:**
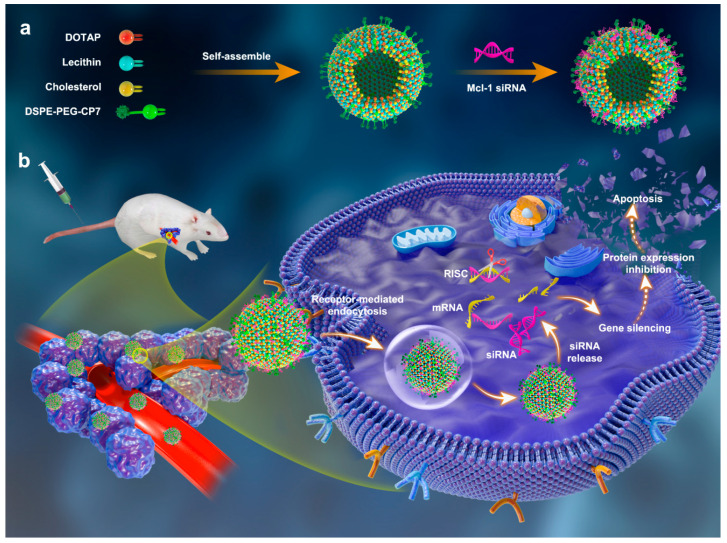
Schematic illustration of (**a**) formation of CP7-modified cationic liposomes and (**b**) CP7-modified cationic liposomes-mediated efficient siRNA delivery.

Hence, in this study, a safe, targeted, and efficient gene delivery system consisting of target molecule-vector-siRNA was designed. Firstly, CP7-modified DSPE-PEG is used to form cationic liposomes with DOTAP and cholesterol, endowing the system with good biocompatibility and biodegradability, as well as active targeting capabilities. Subsequently, Mcl-1 siRNA (siMcl-1) is encapsulated through electrostatic interaction between anionic siRNA and cationic liposomes to assemble a nanocomplex capable of protecting siMcl-1 from nuclease degradation in serum. All results demonstrated that this delivery system could efficiently transfect therapeutic genes into target cells and induce apoptosis in vitro and also showed good biocompatibility and tumor suppression effects in in vivo experiments, indicating its potential as an excellent nanocarrier for further application in gene therapy (Scheme 1).

## 2. Results and Discussion

### 2.1. Docking Study

**Figure 1:**
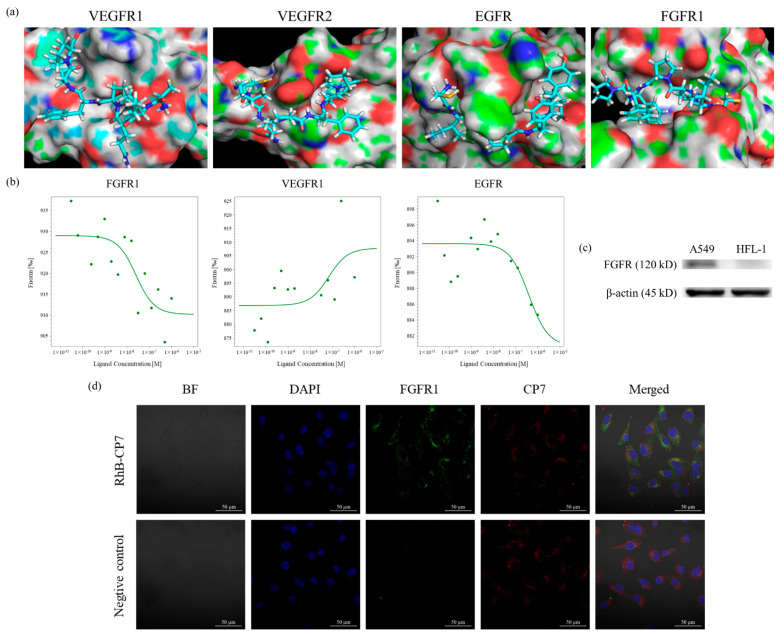
(**a**) Visualization of binding modes within active sites of VEGFR1, VEGFR2, EGFR, and FGFR1. CP7 is shown in stick representation in the binding pocket of selected targets, and proteins are represented using surface model. (**b**) Interaction between CP7, FGFR1, VEGFR1, and EGFR detected by MST. (**c**) Level of FGFR1 protein expressed in A549 and HFL-1 cells determined with Western blot technique. (**d**) Immunofluorescence colocalization of CP-7 peptide with an antibody specific for FGFR1. Scale bar = 50 μm.

Glide in the Schödinger 2018 software was applied to virtual docking, and SiteMap was applied to the search for protein binding sites. The binding between VEGFR1, VEGFR2, EGFR, FGFR1, and CP7 was demonstrated (Figure 1a), and the binding of VEGFR3 with CP7 was demonstrated in our previous work. It was found that compared with VEGFR1, VEGFR2, and EGFR, FGFR1 with a deep pocket was more closely combined with CP7. The hydrogen bond interaction between selected proteins and CP7 was further analyzed and is shown in Figure S1. As shown in Figure S1, the amino and sulfhydryl groups of cysteine residues, as well as amino groups of glutamine in CP7, form three hydrogen bonds with Glu274 in FGFR1 protein. The tyrosine and cysteine residues in CP7 form two hydrogen bonds with Arg202 and Gly204, respectively. Taking the Glide score and hydrogen bond interaction into consideration, FGFR1 was considered to be a good action target for CP7. In order to verify the results of molecular docking, we tested the binding ability of the CP7 peptide to the selected proteins by the microscale thermophoresis (MST) method (Figure 1b). The results showed that the dissociation constants (KD) of the CP7 peptide with FGFR1, VEGFR1, and EGFR were 22.5, 66.1, and 389 nM, respectively, indicating that CP7 has the strongest affinity for FGFR1, which suggests that CP7 can serve as a superior FGFR1-binding peptide.

### 2.2. Study on FGFR1-Mediated Binding of CP7 and A549 Cells

In order to further verify that FGFR1 is also a receptor that the CP7 peptide can bind well to A549 cells, we first explored the level of FGFR1 protein expressed in A549 and HFL-1 cells. From the results of Western blot in Figure 1c, it can be seen that the expression of FGFR1 on A549 cells was much higher than that of normal HFL-1 lung cells, which shows that FGFR1 could indeed be used as a targeted receptor to achieve precise drug delivery to tumor cells. Subsequently, we coincubated the rhodamine-B-labeled CP7 peptide (red fluorescence) with A549 cells and performed immunofluorescence staining on FGFR1 (stained with a fluorescent secondary antibody labeled with CL488, green fluorescence). It can be seen from the results of the laser confocal microscope (Figure 1d) that there was a large amount of green fluorescence on A549 cells, while there was almost no green fluorescence in the negative control group (without the addition of FGFR1 primary antibody), indicating that the expression level of FGFR1 is indeed high. At the same time, a lot of red fluorescence could be seen on the cells, indicating that the CP7 peptide can bind well to A549 cells. In addition, the overlap rate of green fluorescence and red fluorescence is very high, which proves that FGFR1 could indeed mediate the binding of the CP7 peptide to A549 cells.

### 2.3. Synthesis and Characterization of DSPE-PEG/DSPE-PEG-CP7

**Figure 2:**
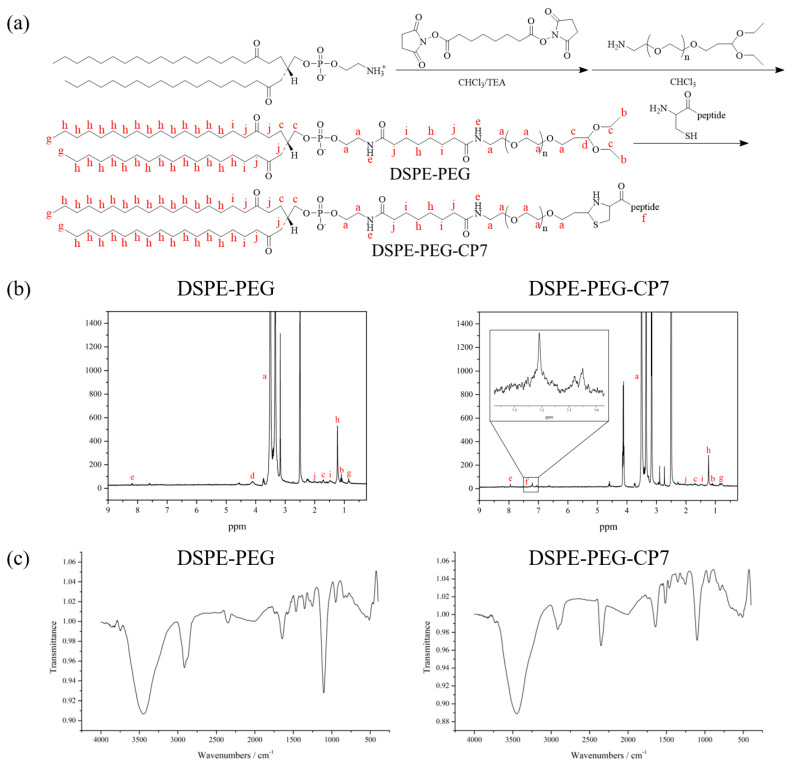
Synthetic route (**a**), ^1^H−NMR spectra (**b**), and FT−IR spectrum (**c**) of DSPE-PEG and DSPE-PEG-CP7.

The structure of DSPE-PEG and DSPE-PEG-CP7 was confirmed by ^1^H-NMR and FT-IR after purification. The ^1^H-NMR spectra of DSPE-PEG in Figure 2b shows the peaks around 3.51 ppm (a) were attributable to the protons (-CH_2_CH_2_O) in the PEG chain. The peaks around 1.10 (b), 1.72 (c), and 4.11 ppm (d) were attributable to the protons ((CH_3_CH_2_O)_2_CH-) in the acetal group. The peaks around 8.20 ppm (e) were attributable to the protons (-CO-NH), which indicate the successful synthesis of DSPE-PEG. The spectra of DSPE-PEG-CP7 are shown in Figure 2b. It can be seen that the characteristic peaks of the PEG chain and -CO-NH still existed. Meanwhile, the peaks around 7.6 ppm (f) were attributed to the protons of tyrosine in the CP7 peptide, providing evidence of the connection between CP7 and DSPE-PEG. In addition, 0.8 ppm (g) was attributable to the protons in the methyl group (-CH_3_) at the DSPE end, 1.26 ppm (h) was attributable to the protons in the methylene group (-CH_2_CH_2_CH_2_-) on the aliphatic chain, 1.53 ppm (i) was attributable to the protons in -CH_2_CH_2_CO-, and 2.0 ppm (j) was attributable to the protons in -CH_2_CO-. For the FT-IR of DSPE-PEG in Figure 2c, the peaks at 2910 cm^−1^, 1635 cm^−1^, and 1097 cm^−1^ belong to -CHO, -C=O, and -C-O-C, respectively, and the peak at 3450 cm^−1^ is attributable to -NH. Meanwhile, in the FT-IR of DSPE-PEG-CP7 (Figure 2c), in addition to the characteristic peaks that appeared in DSPE-PEG, a new peak at 1506 cm^−1^ that belonged to benzene of tyrosine in the CP7 peptide also arose. All the characteristic peaks in the ^1^H-NMR and FT-IR spectra above confirm the successful synthesis of DSPE-PEG and DSPE-PEG-CP7.

### 2.4. Preparation and Characterization of Liposomes

**Figure 3:**
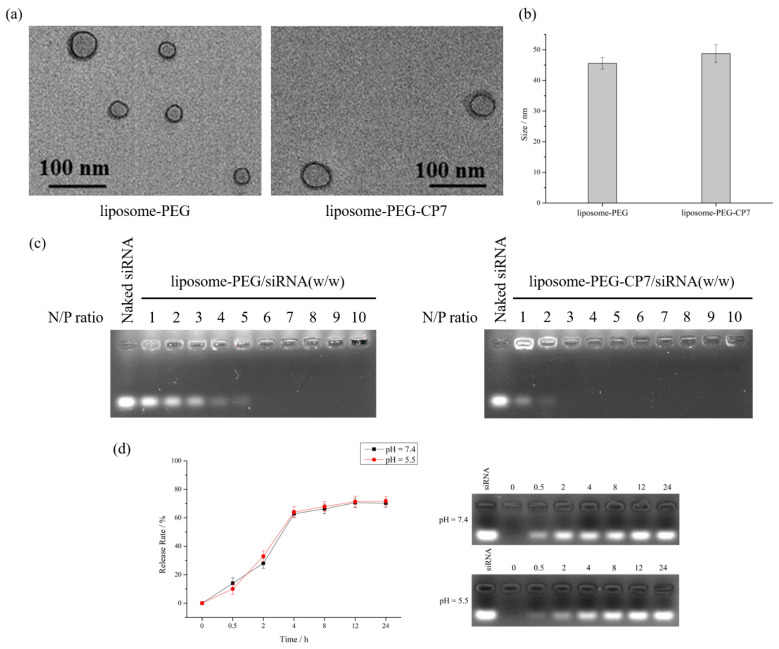
(**a**) TEM images of liposome-PEG and liposome-PEG-CP7. (**b**) Size distribution of liposome-PEG and liposome-PEG-CP7 by dynamic light scattering (DLS) analysis. Data are presented as means ± SD (n = 3). (**c**) siRNA binding ability of liposome-PEG and liposome-PEG-CP7 evaluated by agarose gel retardation assay. (**d**) Drug release profiles of liposome-PEG-CP7 in PBS at different pH. The right panel is a typical result of drug release assayed by agarose gel electrophoresis.

After the successful synthesis of DSPE-PEG and DSPE-PEG-CP7, we prepared liposome-PEG and liposome-PEG-CP7 through a film dispersion method by mixing them with another lipid material. The surface charge of liposomes was investigated immediately by the Malvern Zetasizer Nano ZS 90 zeta potential analyzer. It is worth noting that liposome-PEG showed a positive surface charge of +26.6 mV, whereas the surface charge of liposome-PEG-CP7 increased to +36.1 mV due to the overlay of positive charge from CP7 molecules, which can combine the PEG terminus by a chemical bond. The increasing positive potential was favorable for subsequent cell uptake and drug delivery. Furthermore, the morphology observed by TEM of liposome-PEG and liposome-PEG-CP7 was in shape, with a diameter of about 50 nm (Figure 3a), and peptide ligand modification resulted in a slight increase in liposomal particle size, which is consistent with the results (Figure 3b) of dynamic light scattering (DLS). In addition, for there is no sulfur element in the liposome-PEG sample, we used ICP-MS to characterize the conjunction of the CP7 peptide. The results obtained by ICP-MS showed 49.7 mg/L sulfur from CP7 peptide molecules in liposome-PEG-CP7, indicating a coupling ratio of 45.04%.

### 2.5. Loading and Release of siRNA

The siRNA with a negative charge could be loaded on cationic liposomes through electrostatic interaction. siMcl-1 was chosen as the therapeutic gene for drug loading. Owing to the abundant cations on both liposome-PEG and liposome-PEG-CP7, the efficient loading of siMcl-1 was achieved after mixing siRNA with the liposomes. Then we investigated the optimal N/P ratio of the siRNA/lipid complex by agarose gel electrophoresis, in which the siRNA/lipid complex was blocked in the original groove, while unloaded siRNA could move in the positive direction at a certain rate. As expected, the naked siRNA was completely swept out of the sample hole under the action of an electric field, moving in the positive direction with obvious bands appearing. Moreover, a large number of siRNAs were released from the gel pores under the action of an electric field force at an N/P ratio of 1 and 2, indicating that the binding of siRNA to the complex was not tight enough, and the complex was not stable in case of these N/P ratios. As the N/P ratio increased, the binding between siRNA and complexes became gradually tight, and it can be found that almost all of the siRNA was blocked in the gel pore, and the free siRNA band disappeared when the N/P ratio was 6, demonstrating that stable complex was formed, and the siRNA completely encapsulated by liposome-PEG (Figure 3c). Similarly, the free siRNA band gradually disappeared as the N/P ratio increased, while almost all of the siRNA was encapsulated and blocked at the origin groove through the loading effect by liposome-PEG-CP7 at the N/P ratio of 6. Therefore, the optimal N/P ratios of siRNA/liposome-PEG and siRNA/liposome-PEG-CP7 were 6 and 3, which are also consistent with the conclusion that the gene loading capacity of the vector is positively correlated with its positive charge. The in vitro release experiment (Figure 3d) demonstrated that the siRNA in siRNA/liposome-PEG-CP7 could be effectively released within 4 h so that the siRNA in the nanoparticles could function effectively after reaching the target cells. At the same time, there was no statistically significant difference in the release behavior under the two pH conditions, which coincides with the design of our lipid material without modification of sensitive groups.

### 2.6. In Vitro Cell Cytotoxicity Research

**Figure 4:**
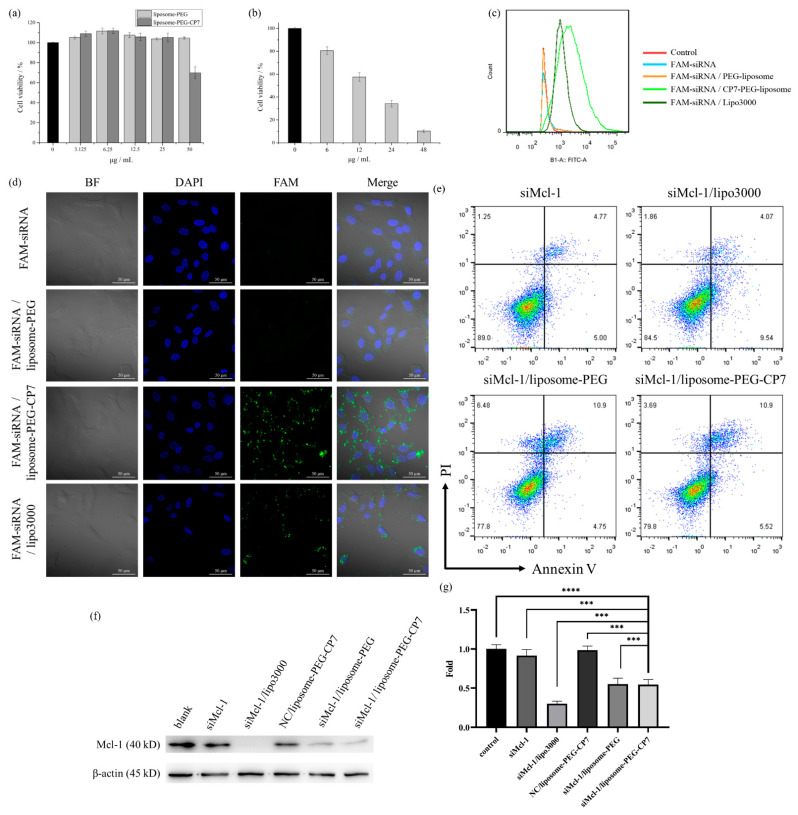
(**a**) Cytotoxic effect of liposome-PEG and liposome-PEG-CP7 on HFL-1 cells. (**b**) Cytotoxic effect of siMcl-1/liposome-PEG-CP7 on A549 cells. (**c**) Cell uptake of siRNA/liposomes on A549 cells determined with FCM. (**d**) Cell uptake and intracellular distribution of siRNA/liposomes in A549 cells determined with CLSM. (**e**) Apoptosis induction by siRNA/liposomes in A549 cells determined with FCM. (**f**) Level of Mcl-1 protein expressed in A549 cells treated with siRNA/liposomes determined with Western blot technique. (**g**) Level of Mcl-1 mRNA expressed in A549 cells treated with siRNA/liposomes determined with RT−PCR. *: differences between siMcl-1/liposome-PEG-CP7 with other groups, ***: *p* < 0.001, ****: *p* < 0.0001.

It is well known that the cytotoxicity of carriers is the primary consideration we should pay attention to. Hence, we evaluated the cytotoxicity of liposome-PEG and liposome-PEG-CP7 against normal HFL-1 cells by MTT assay firstly. Figure 4a shows that at all concentrations, liposome-PEG showed no significant toxicity to HFL-1 cells, and the cell viability was nearly 100%. For liposome-PEG-CP7, cell viability also reached 70% at 50 μg/mL. These results indicated that the liposome-PEG and liposome-PEG-CP7 are both relatively safe as suitable drug delivery vehicles. To directly confirm the therapeutic potential of siMcl-1/liposome-PEG-CP7, the cytotoxicity assay reflecting cell metabolism inhibition of the complex against A549 was performed. It can be seen from Figure 4b that the complex has good inhibitory activity on A549 cells after 24 h of treatment, with an IC_50_ of 14.94 μg/mL. For further in vitro studies, we used liposome-PEG at a concentration of 27.6 μg/mL and liposome-PEG-CP7 at 12 μg/mL.

### 2.7. Liposome-PEG-CP7 Increased Cellular Uptake of A549 Cells

To verify the specificity/selectivity of the CP7-based targeting system, flow cytometric (FCM) analyses of siRNA/liposome-PEG-CP7 in A549 cell lines were carried out. Results showed that the siRNA/liposome-PEG-CP7 were carried into the A549 cells at the highest intensity of FITC fluorescence compared with siRNA/PEG-liposome, siRNA control group, and positive group (siRNA/lipo3000), respectively (Figure 4c). Confocal laser scanning microscopy (CLSM) imaging was further used to trace the intracellular distribution of the siRNA/liposome-PEG-CP7 system in A549 cells after incubation for 4 h. As shown in Figure 4d, the single FAM-siRNA was rarely distributed inside the cell because such a small molecule could not easily permeate the cell membranes. When siRNA was delivered by liposome, compared with nontargeted siRNA/liposome-PEG system whose internalization in cells was similar to single siRNA, the targeted siRNA/liposome-PEG-CP7 mainly localized in the cytosol, and dot-shaped fluorescence with a higher intensity than the positive control (siRNA/lipo3000) could be observed in the cytoplasm. The reason for this phenomenon was the specific binding between CP7 with A549 cells, which increased the chance of the targeted liposome system to enter the cell through endocytosis. Therefore, the CP7 peptide could be targeted to A549 cells and subsequently improved the internalization of the corresponding liposome system. We then tested the zeta potential of the NC/liposome complexes formed under the optimal N/P. The results showed that the zeta potential values of NC/liposome-PEG and NC/liposome-PEG-CP7 were +15.8 and +16.7 mV, respectively, indicating that the potential difference between the two NC/liposome complexes is small under the optimal N/P. Taken together, these results clearly demonstrated that siRNA/liposome-PEG-CP7 could efficiently target A549 cells by the high specificity of CP7.

### 2.8. Liposome-PEG-CP7 Promoted Apoptosis of A549 Cells

Flow cytometry analysis was further applied for evaluating the apoptotic-induced activity of siRNA/liposome-PEG-CP7 after being internalized by A549 cells. As shown in Figure 4e, the apoptosis rate of A549 cells treated with siRNA/liposome-PEG-CP7 reached up to 16.42%, which was better than the siRNA/lipo3000 positive control (13.61%), whereas the single Mcl-1 siRNA had lower apoptotic-induced activity as 9.77%.

For a deeper investigation relevant to factors of tumor cell apoptosis and to examine the biological activities of siRNA after treatment, Western blot analysis of Mcl-1 protein from the A549 tumor cells was performed (Figure 4f). After incubating A549 cells with different samples for 24 h, there was almost no Mcl-1 protein expression in the siRNA/lipo3000 group, which was attributed to the strong gene transfection ability of lipo3000. At the same time, the band of the Mcl-1 protein of siMcl-1/liposome-PEG-CP7 was also very shallow, indicating that our nanocarrier can effectively transfect siMcl-1 into A549 cells and silence the expression of the corresponding protein. In contrast, in the siMcl-1 and NC/liposome-PEG-CP7 groups, the Mcl-1 protein band was similar to the blank group, indicating that it is difficult for siMcl-1 to enter cells alone to generate gene silencing, while liposome-PEG-CP7 also cannot reduce the expression of Mcl-1 protein. All these indicated that liposome-PEG-CP7 can effectively transfect siMcl-1 into A549 cells and induce gene silencing.

At the same time, total RNA from A549 cells was extracted after interacting with various samples, and the messenger RNA (mRNA) transcription level of the Mcl-1 gene was detected by RT-PCR (Figure 4g). The siMcl-1/lipo3000 group also showed the lowest Mcl-1 mRNA transcription level (about 0.30 times that of the blank group). The inhibitory effect of the siMcl-1/liposome-PEG-CP7 group on Mcl-1 mRNA transcription followed closely, which was about 0.54 times that of the blank group. Consistent with the results of Western blotting, neither the NC/liposome-PEG-CP7 group nor the siMcl-1 group showed a significant inhibitory effect on the transcription of Mcl-1 mRNA. All these indicate that liposome-PEG-CP7 can effectively transfect siMcl-1 into A549 cells and induce gene silencing.

### 2.9. In Vivo Antitumor Activity and Toxicity Assay

**Figure 5:**
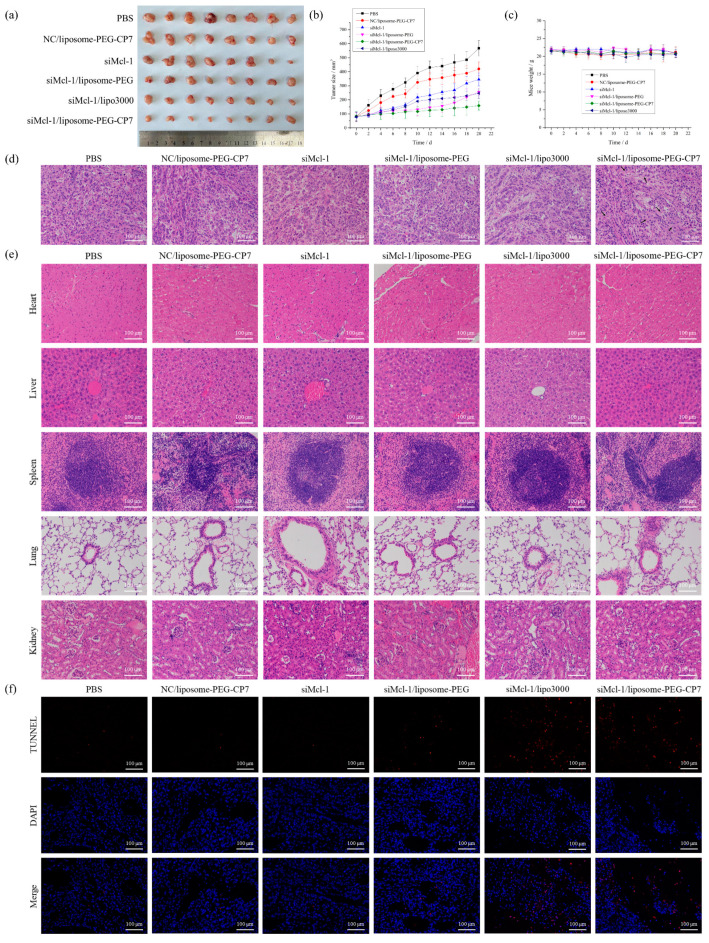
(**a**) Photo of tumor tissues from mice treated with PBS, siMcl-1/lipo3000, siMcl-1/liposome-PEG-CP7, siMcl-1/liposome-PEG, NC/liposome-PEG-CP7, and free siMcl-1 at the end of the study. (**b**) Real-time observation of tumor sizes in vivo after treatment with samples. Error bars represent means ± SD. (**c**) Real-time weight analysis of mice after each treatment. Error bars represent means ± SD. (**d**,**e**) H&E staining of tumor tissue (**d**) and major organs (heart, liver, spleen, lung, and kidney) (**e**) after treatment. (**f**) TUNEL staining of tumor tissue after treatment.

The preliminary tumor inhibition effect of siMcl-1/liposome-PEG-CP7 was investigated on nude mice bearing the subcutaneous A549 tumors. PBS, siMcl-1/lipo3000, siMcl-1/liposome-PEG-CP7, siMcl-1/liposome-PEG, NC/liposome-PEG-CP7, and free siMcl-1 were intravenously injected into these mice every 3 days, respectively, and the inhibition efficacy toward tumor growth was monitored. The dose of siRNA was 2.5 mg/kg, and the corresponding doses of liposome-PEG and liposome-PEG-CP7 were 134.58 mg/kg and 58.18 mg/kg, respectively. The tumor tissues separated from different treatment groups after treatment for 20 days are shown in Figure 5a. As shown in Figure 5b, the tumors treated with the control saline grew rapidly, which exhibited the typical growth behavior of the tumor. At the same time, siMcl-1 and liposome-PEG-CP7 alone had a less inhibitory effect on growth, and the growth behavior of tumors was not significantly different from that of the PBS group. In comparison, the therapeutic effect was obviously improved, when siMcl-1 was loaded with lipo3000 and liposome-PEG. As expected, all results indicated the siRNA/liposome-PEG-CP7 group performed better curative effects than other groups studied over the three-week period. In addition, during the experiment period of 20 days, these mice did not show obvious variation in their body weights (Figure 5c). The results suggested that siRNA/liposome-PEG-CP7 had good biocompatibility and no obvious toxicity for the mice.

Furthermore, in order to assess the efficiency of gene therapy, H&E staining was performed to observe the morphologies of the tumor cells (Figure 5d). Most tumor cells in the saline and free siMcl-1 groups exhibited normal morphologies. On the contrary, tumor cells in the siMcl-1/liposome-PEG-CP7 group showed obvious apoptosis morphologies such as nucleus shrinkage. This is consistent with the results of TUNEL staining (Figure 5f). Compared with other groups, the highest density of red fluorescence was observed in the siMcl-1/liposome-PEG-CP7 group, indicating that a lot of cell apoptosis occurred after administration. These results illustrated that owing to the modification of targeting peptide CP7, the siMcl-1 had been precisely delivered by liposome into tumor cells to induce cell apoptosis and inhibit tumor growth.

In addition, H&E staining was also performed on the major organs such as the heart, liver, spleen, lung, and kidney, as displayed in Figure 5e. Neither histopathological abnormalities nor damage were apparent after intravenous injection, suggesting that this peptide ligand-mediated liposome system for targeted delivery of gene drug is safe enough in vivo.

## 3. Materials and Methods

### 3.1. Chemicals and Apparatus

All reagents and solvents were commercially available and used without additional treatment. Distearoyl phosphoethanolamine (DSPE) and (2,3-dioleoyloxy-propyl)-trimethylammonium (DOTAP) were purchased from Shanghai Advanced Vehicle Technology Co., Ltd (China). Disuccinimidyl suberate (DSS), phospholipid, and cholesterol were obtained from Aladdin (China). CP7 and rhodamine-B-conjugated CP7 were synthesized by Shanghai GL Biochem Peptide Ltd. (China). Negative control siRNA (NC siRNA), fluorescence-labeled siRNA (FAM-siRNA), siMcl-1 and the primer of β-actin and Mcl-1 were purchased from GenePharma (Shanghai, China). Dulbecco’s modified Eagle’s medium (DMEM), fetal bovine serum (FBS), 3-(4,5-dimethylthiazol-2-yl)-2,5-diphenyltetrazolium bromide (MTT), trypsin/EDTA, penicillin/streptomycin, dimethylsulfoxide (DMSO), 4,6-diamidino-2-phenylindole (DAPI), and Apoptosis Kit were obtained from Gibco (USA). Human FGFR1/CD331 protein (His tag) (10616-H08H), human VEGFR1/FLT-1 protein (Fc tag) (10136-H02H), and human EGFR/HER1/ErbB1 protein (His tag) (10001-H08H) were purchased from Sino Biological (China). Radioimmunoprecipitation (RIPA) lysis buffer and phenylmethanesulfonyl fluoride (PMSF) were purchased from Beyotime Biotechnology (China). β-actin rabbit antibody (#4970), Mcl-1 rabbit antibody (#5453), FGFR1 rabbit antibody (#9740), and goat anti-rabbit IgG HRP-linked antibody (#7074) were purchased from Cell Signaling (USA). Goat anti-rabbit IgG(H + L) and CoraLite488 conjugate (SA00013-2) were purchased from Proteintech (USA). Trizol reagent was purchased from Invitrogen (USA). PrimeScriptRT Master Mix and SYBR Premix Ex Taq II were purchased from Takara (Japan). Other reagents and chemicals were of at least analytical reagent grade.

### 3.2. Characterization

Microscale thermophoresis analysis was performed with a Monolith NT.115 (Germany). Fourier-transform infrared spectroscopy (FT-IR) (4000–400 cm^−1^) was performed on a Bruker FT-IR spectrometer (Germany) using KBr pellets with a resolution of 2.0 cm^−1^. ^1^H-NMR spectra were obtained using Bruker Avance-300 and Bruker Avance-500 instruments (Germany) calibrated to DMSO-d6 as the internal reference. Transmission electron microscopy (TEM) was performed on a JEOL 2100 (Japan) with an accelerating voltage of 200 kV. TEM samples were prepared by drop-casting dispersion in ethanol solution onto copper grids covered by carbon film and dried at ambient temperature. Size distribution and surface charge of the nanocarrier were investigated on a Malvern Zetasizer Nano ZS 90 zeta potential analyzer (UK). The determination of sulfur content was performed with inductively coupled plasma-mass spectrometry (ICP-MS, Optima 5300DV, PerkinElmer, Waltham, MA, USA). Confocal images were acquired using a Zeiss confocal laser scanning unit mounted on an LSM 710 fixed-stage upright microscope (Germany). Flow cytometry experiments were performed with a BD FACSAria apparatus (USA).

### 3.3. Docking Studies

The crystal structures of VEGFR1 (PDB Code: 5T89), VEGFR2 (PDB Code: 5OYJ), VEGFR3 (PDB Code: 4BSJ), EGFR (PDB Code: 4KRL), and FGFR1 (PDB Code: 1EVT) were downloaded from the RCSB Protein Data Bank. The receptor proteins were then optimized using the Protein Preparation Wizard workflow in Schödinger 2018 (Schödinger, New York, USA), which involves removing water molecules, unrelated ligands and ions, and single-strand proteins from the protein’s crystalline structure. The structure of the ligand (CP7) was drawn with ChemDraw 19.0 (PerkinElmer, Waltham, MA, USA), converted into a 3D structure, and saved as MDL SDF files in ChemDraw 19.0. The LigPrep workflow was used for preparation of the ligand molecules (force field: OPLS 2005), and the resulting structure was saved in Maestro format for molecular docking. The sitemap of Schödinger 2018, using water molecules as probes, was used to predict the putative binding site of receptor proteins, which was based on scores of van der Waals and electrostatic interactions between molecular probes and protein surface atoms. Receptor grid generation was applied to construct the docking grid files, and ligand docking was applied for molecule docking. Finally, a favorable protein target was identified by hydrogen bond interactions between ligand and receptor protein and scores of ligand in selected receptor-protein-specified binding sites.

### 3.4. Microscale Thermophoresis Analysis

The protein was dissolved in PBS solution containing 0.05% Tween-20 and then prepared into a series of predesigned gradient dilutions. An equal amount of the labeled peptide to be tested was added and mixed thoroughly. To analyze the thermophoresis of a sample, 10 μL was transferred to a glass capillary and analyzed at room temperature.

### 3.5. Cell Culture

The lung carcinoma cell (A549 cell) and human normal embryonic lung fibroblast cell (HFL-1 cell) were provided by Key-GEN Biotech and maintained in DMEM containing 10% FBS, 100 units/mL penicillin, and 0.1 mg/mL streptomycin in a 5% CO_2_ incubator at 37 °C.

### 3.6. Immunofluorescence Staining

For immunofluorescence staining, 5 × 10^4^/well of A549 cells were seeded in 35 mm dishes with a glass bottom and incubated overnight in a 5% CO_2_ incubator at 37 °C. Then, cells were washed with PBS and treated with rhodamine-B-conjugated CP7 for 4 h. After washing with PBS three times, the adherent cells were fixed with 500 μL 4% paraformaldehyde for 20 min. Then, cells were blocked with 10% normal goat serum in 1% PBS, followed by overnight incubation at 4 °C with primary antibodies/mouse anti-FGFR1 (1:200 dilution). After washing with PBS, cells were incubated with the CL488-conjugated secondary antibody (1:200 dilution) for 1 h. The nuclei were stained with DAPI for 15 min, and fluorescent images were recorded by confocal laser scanning microscopy.

### 3.7. Syntheses of DSPE-Polyethylene Glycol (PEG)

The synthesis of acetal-PEG-NH_2_ referenced the previous work by our group [21]. The activation of the amino group on DSPE was carried out by the following method: The mixture of 20.3 mg DSPE and 16.3 mg DSS was dissolved in 5 mL CHCl_3_, then 20 μL TEA was added to the mixture. Subsequently, the mixture was stirred overnight at room temperature under the protection of N_2_. Finally, DSPE was activated to DSPE-CO-(CH_2_)_6_-CO-NHS at the end of the reaction. After that, 80 mg acetal-PEG-NH_2_ was dissolved in CHCl_3_ and then dropped into the previously activated reaction solution. Then, the mixture was stirred for 24 h at room temperature in the dark. CHCl_3_ was removed by evaporation to obtain the crude product, then the product was redissolved by distilled water and poured into a dialysis bag (MWCO = 8000–14,000 Da) with an external solution of Na_2_CO_3_ for 24 h and distilled water for another day. Finally, the mixture was freeze-dried, and pure DSPE-PEG was obtained (yield: 72%). The structure of DSPE-PEG was confirmed by ^1^H-NMR and FT-IR.

### 3.8. Syntheses of DSPE-PEG-CP7

CP7 and DSPE-PEG were mixed in AcOH (0.2 mol/L, pH 4.0), then the mixture was stirred at room temperature for 5 days. Next, it was poured into a dialysis bag (MWCO = 3500 Da) with an external solution of distilled water. Finally, the mixture was freeze-dried, and pure DSPE-PEG-CP7 was obtained (yield: 79%). The structure of DSPE-PEG-CP7 was confirmed by ^1^H-NMR and FT-IR.

### 3.9. Preparation of Liposome-PEG-CP7 and Liposome-PEG

To prepare liposome-PEG-CP7 and liposome-PEG, the lipid mixture of lecithin, DOTAP, cholesterol, and DSPE-PEG or DSPE-PEG-CP7 at a molar ratio of 4:4:2.5:1 was dissolved in ethanol and then dried under a rotary evaporator to obtain a homogeneous lipid membrane, which needed to be vacuum-dried for 24 h. Afterward, the dried lipid film was hydrated with phosphate-buffered saline (PBS, pH 6.86) and stirred at room temperature overnight, followed by being sonicated and extruded through a 200 nm polycarbonate filter to reduce the particle size. Finally, the liposome dispersion was freeze-dried for further use.

### 3.10. siRNA Loading and Release

To determine the optimal N/P ratio between siRNA with liposome-PEG-CP7 or liposome-PEG, 1 μL siMcl-1 (0.64 nmol/μL) was mixed with lipid dispersion at different concentrations, then the mixtures were placed for 20 min to form the siRNA/lipid complex. Free siMcl-1 was used as a control group. Agarose gel retardation assay was used to determine the loading and the optimal N/P ratio of siRNA by different lipid complexes. The nucleic acid bands were visualized by UV imaging equipment (ABI, GIS-2500).

The release of siRNA/liposome-PEG-CP7 at different pH was performed according to the literature [28]. Then, 2 μL siMcl-1 (0.64 nmol/μL) was mixed with liposome-PEG-CP7 dispersion to achieve N/P 3, and the final volume was adjusted to 10 µL by phosphate buffer (pH 7.4 or 5.5). After incubation at 37 °C on an orbital shaker for a pre-set time, all samples were then subjected to agarose gel electrophoresis. Nucleic acid bands were visualized and analyzed for relative quantification by UV imaging equipment (ABI, GIS-2500).

### 3.11. Cell Cytotoxicity Assay

The cell cytotoxicity of liposome-PEG-CP7 and liposome-PEG on HFL-1 cells and siMcl-1/liposome-PEG-CP7 complex on A549 was determined quantitatively by MTT assay. In a typical procedure, cells were initially seeded into a 96-well plate at a density of 1 × 10^4^ cells/well and incubated for 24 h at 37 °C in a humidified atmosphere containing 5% CO_2_. These cells were, respectively, incubated with liposome-PEG-CP7, liposome-PEG, and siMcl-1/liposome-PEG-CP7 complex with various concentrations for 24 h under the same conditions. After the incubation, cells were washed three times with 200 μL PBS to remove the unbound drugs. Subsequently, fresh culture medium (200 μL) containing MTT solution (20 μL, 5 mg/mL) was added to each well and then incubated for another 4 h. Finally, the incubated medium was removed, and 150 μL DMSO was added to each well to dissolve formazan crystals, and they were then gently shaken for 10 min at room temperature. Absorbance of the solution was measured by enzyme-linked immunosorbent assay (ELISA) at a wavelength of 490 nm. The absorbance value of untreated cells was set at 100%. Each experiment was repeated three times in six replicates.

### 3.12. Investigation of Cellular Uptake

A549 cells were cultured into a 6-well plate at an initial seeding density of 3 × 10^5^ cells/well. After being treated with FAM-siRNA, FAM-siRNA/liposome-PEG, FAM-siRNA/liposome-PEG-CP7, and FAM-siRNA/lipo3000 at 37 °C for 4 h, cells were collected, washed, and resuspended in PBS. The percentage of cells internalized with different samples was analyzed by flow cytometry. The siRNA/lipid complexes for assay were all at an optimal N/P ratio. The concentrations of siRNA in all samples were equal.

### 3.13. Investigation of Subcellular Location

For investigation of the subcellular location, 5 × 10^4^/well of A549 cells were seeded in 35 mm dishes with a glass bottom and incubated overnight in a 5% CO_2_ incubator at 37 °C. Then, cells were washed with PBS and treated with FAM-siRNA, FAM-siRNA/liposome-PEG, FAM-siRNA/liposome-PEG-CP7, and FAM-siRNA/lipo3000. After culturing for 4 h, cells were washed with PBS three times and mixed with 500 μL 4% paraformaldehyde for 20 min. The nuclei were stained with DAPI for 15 min, and fluorescent images were recorded by confocal laser scanning microscopy. The siRNA/liposome complexes for assay were all at an optimal N/P ratio. The concentrations of siRNA in all samples were equal.

### 3.14. Cell Apoptosis Assay

A549 cells were cultured in a 6-well plate at an initial seeding density of 3 × 10^5^/well. After being treated with siMcl-1, siMcl-1/liposome-PEG, siMcl-1/liposome-PEG-CP7, and siMcl-1/lipo3000 at 37 °C for 24 h, the cells were collected, washed, and resuspended in PBS, then labeled by annexin V-FITC conjugate and PI. Finally, the percentage of apoptotic cells of different samples was analyzed by flow cytometry. The siRNA/lipid complexes for assay were all at an optimal N/P ratio, and the concentrations of siRNA in all samples were equal.

### 3.15. Western Blotting Assay

To explore the FGFR1 expression of A549 and HFL-1, cells were grown to 70–80% confluence. In order to explore the effect of siRNA/liposome complexes on Mcl-1 protein expression, A549 cells at a density of 3 × 10^5^/well were treated with siMcl-1, siMcl-1/liposome-PEG, siMcl-1/liposome-PEG-CP7, and siMcl-1/lipo3000 at 37 °C for 24 h. After that, cells were washed with PBS three times, and the proteins were extracted by RIPA lysis buffer supplemented with protease inhibitor cocktail and 1 mM PMSF. Then, equal amounts of proteins were added to SDS-PAGE gels and separated by gel electrophoresis. After transferring the proteins from gel to polyvinylidene difluoride (PVDF) membrane, the blots were blocked with 3% BSA in TBST and then incubated with FGFR1 (1:1000 dilution) or Mcl-1 rabbit antibody (1:1000 dilution) and β-actin rabbit antibody (1:1000 dilution). The FGFR1 or Mcl-1 expression was detected with an HRP-conjugated secondary antibody (1:1500 dilution) and an enhanced chemiluminescence (ECL) detection system (Tanon). The siRNA/lipid complexes for assay were all at an optimal N/P ratio, and the concentrations of siRNA in all samples were equal.

### 3.16. Gene Silencing Assay

A549 cells were seeded into a 6-well plate at 3 × 10^5^/well and incubated for 24 h at 37 °C. After treatment with siMcl-1, siMcl-1/liposome-PEG, siMcl-1/liposome-PEG-CP7, and siMcl-1/lipo3000 for 24 h, total RNA was extracted using Trizol reagent according to the manufacturer’s procedure. The cDNA of β-actin and Mcl-1 was generated using PrimeScriptRT Master Mix and detected with real-time PCR (RT-PCR) to calculate the level of cellular β-actin and Mcl-1 mRNA. Each experiment was repeated three times in three replicates. The primer sequences used for qPCR are listed as follows.

β-actin: AAACGTGCTGCTGACCGAG(F)

TAGCACAGCCTGGATAGCAAC(R)

Mcl-1: CGCCAAGGACACAAAGCCAAT(F)

CCCGTCGTAAGGTCTCCAGC(R)

### 3.17. Antitumor Effect in A549-Bearing Nude Mice

All animal experiments were performed in accordance with the Guidelines for Care and Use of Laboratory Animals of China Pharmaceutical University and approved by the Animal Ethics Committee of China Pharmaceutical University, Nanjing, China. For the mouse subcutaneous tumor model, 2 × 10^6^ A549 cells (suspended in 200 μL sterile PBS)/mouse were injected into the right hind thigh of BALB/c male nude mice of five to six weeks old. The size of subcutaneous tumors was measured using calipers, and tumor volume was calculated with the following formula: tumor volume = length × width^2^ × 0.5. When the tumor average volume reached 50–100 mm^3^, the mice were randomly assigned into six groups (n = 8 in each group) and intravenously treated with PBS (100 μL), siMcl-1/lipo3000 (100 μL, 1 mg/mL in saline), siMcl-1/liposome-PEG-CP7 (100 μL, 1 mg/mL in saline), siMcl-1/liposome-PEG (100 μL, 1 mg/mL in saline), NC/liposome-PEG-CP7 (100 μL, 1 mg/mL in saline), or free siMcl-1 (100 μL, 1 mg/mL in saline) per mouse. The mice treated with PBS were used as the control group. When PBS and samples were injected, the tumor sizes and mouse weights were measured periodically. The tumors, hearts, livers, spleens, lungs, and kidneys of the mice in all groups were harvested after the treatment for 21 days, fixed in 4% paraformaldehyde solution, embedded in paraffin, and dissected into slices. TUNEL assay was further performed to estimate the apoptosis in the tumor regions. The histological slices were stained with hematoxylin and eosin (H&E) and then observed by a light microscope.

## 4. Conclusions

In this work, we found that FGFR1, like VEGFR3, can serve as an effective binding site for CP7 peptide, which was previously developed by our group to jointly achieve high-efficiency binding to A549 cells. Accordingly, CP7 was applied to construct a peptide ligand-mediated liposome system for targeted delivery of gene drugs. Mcl-1 siRNA that promotes apoptosis to inhibit tumor growth by silencing the related gene could be efficiently loaded into this CP7-modified liposome at an N/P ratio of 6. These NPs can effectively target A549 cells, resulting in an improved internalization. The in vitro apoptosis test showed that the gene delivery system could successfully induce apoptosis through silencing of the designated gene after entering cells. Furthermore, in vivo experiments proved that siRNA/liposome-PEG-CP7 has excellent tumor targeting and tumor inhibition function in tumor-bearing mice. All these properties make the peptide ligand-modified liposome a potential candidate for a safe, targeted, and efficient intelligent delivery system of gene drugs.

## Data Availability

Not applicable.

## Supplementary Materials

The supporting information can be downloaded at: https://www.mdpi.com/article/10.3390/ijms23158380/s1.
