# Supplementary material for: An FGFR1-Binding Peptide Modified Liposome for siRNA Delivery in Lung Cancer
Source: Int J Mol Sci. 2022 Jul 29;23(15):8380. doi: 10.3390/ijms23158380 (PMC9369135; doi:10.3390/ijms23158380)
Supplement: Supplementary file 1 [file ijms-23-08380-s001.zip › ijms-1766705-supplementary.pdf]

Supplementary Information

A FGFR1-binding peptide modified liposome for siRNA delivery in lung cancer

Zhipeng Dong[1], Yunxue Yin[1], Jun Luo[1], Bingxia Li[1], Fangning Lou[1], Qiyan Wang[1], Qingfa Zhou[1], Baofen Ye*[1], Yue Wang*[1]*

1. Key Laboratory of Biomedical Functional Materials, School of Sciences, China Pharmaceutical University, Nanjing 211198, Jiangsu Province, China. E-mail: zwy_1115@126.com

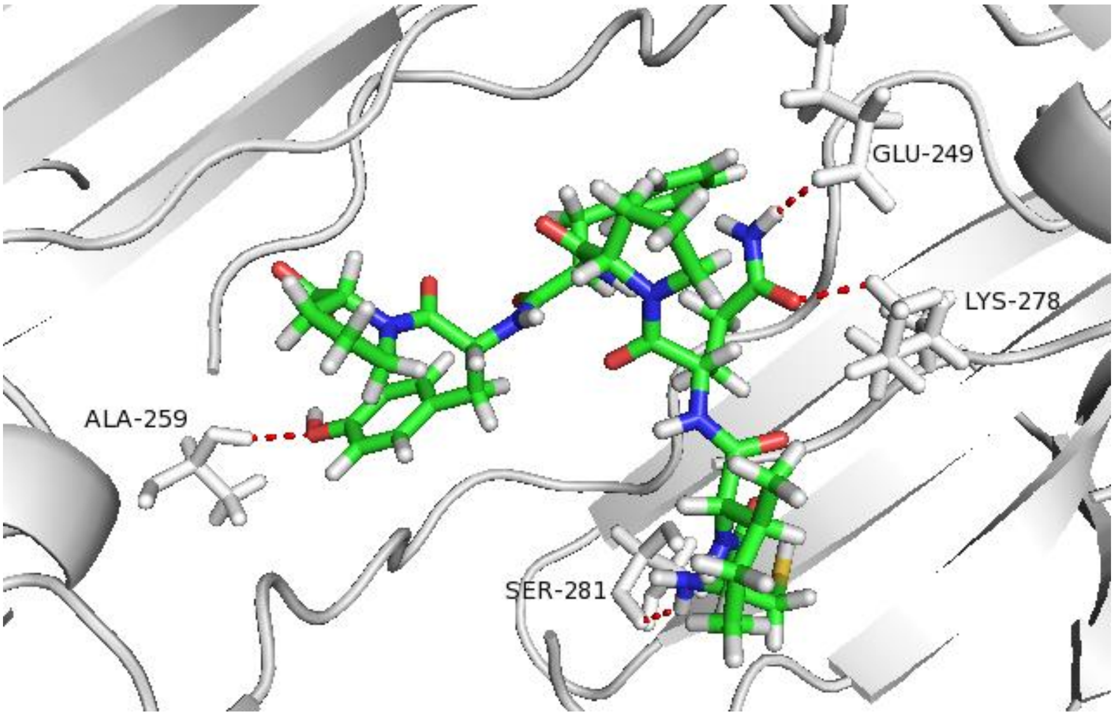

Figure S1. Hydrogen bond interaction of FGFR1 with CP7 and hydrogen bonds are labeled with red lines.
